# Carcinoembryonic antigen (CEA)-related cell adhesion molecules are co-expressed in the human lung and their expression can be modulated in bronchial epithelial cells by non-typable Haemophilus influenzae, Moraxella catarrhalis, TLR3, and type I and II interferons

**DOI:** 10.1186/1465-9921-14-85

**Published:** 2013-08-14

**Authors:** Esther Klaile, Tilman E Klassert, Inka Scheffrahn, Mario M Müller, Annina Heinrich, Kerstin A Heyl, Hendrik Dienemann, Christiane Grünewald, Robert Bals, Bernhard B Singer, Hortense Slevogt

**Affiliations:** 1 Septomics, Research Centre of the Friedrich-Schiller-University Jena, the Jena University Hospital and the Leibniz-Institute for Natural Products Research and Infection Biology – Hans Knöll Institute, Albert-Einstein-Strasse 10, 07745 Jena, Germany; 2 Center for Sepsis Control and Care (CSCC), Jena University Hospital, Erlanger Allee 101, 07747 Jena, Germany; 3 Clinic of Gastroenterology and Hepatology, University Hospital Essen, Hufelandstaße 55, 45147 Essen, Germany; 4 Institute of Microbiology and Hygiene, Charité-Universitätsmedizin Berlin, Hindenburgdamm 27, 12203 Berlin, Germany; 5 Surgical Department, Thoraxklinik, Heidelberg University, Member of German Center for Lung Research, Amalienstraße 5, 69126 Heidelberg, Germany; 6 Department of Internal Medicine V – Pulmonology, Allergology, Respiratory Intensive Care Medicine, University of the Saarland, Kirrbergerstraße 1, 66421 Homburg Saar, Germany; 7 Institute of Anatomy, University Hospital Essen, Hufelandstraße 55, 45147 Essen, Germany

**Keywords:** CEACAM1, CEACAM5 (CEA), CEACAM6, Lung, Normal human bronchial epithelial (NHBE) cells, Interferon, Polyinosinic:polycytidylic acid (poly I:C), Non-typable *Haemophilus influenzae* (NTHI), *Moraxella catarrhalis*

## Abstract

**Background:**

The carcinoembryonic antigen (CEA)-related cell adhesion molecules CEACAM1 (BGP, CD66a), CEACAM5 (CEA, CD66e) and CEACAM6 (NCA, CD66c) are expressed in human lung. They play a role in innate and adaptive immunity and are targets for various bacterial and viral adhesins. Two pathogens that colonize the normally sterile lower respiratory tract in patients with chronic obstructive pulmonary disease (COPD) are non-typable *Haemophilus influenzae* (NTHI) and *Moraxella catarrhalis*. Both pathogens bind to CEACAMs and elicit a variety of cellular reactions, including bacterial internalization, cell adhesion and apoptosis.

**Methods:**

To analyze the (co-) expression of CEACAM1, CEACAM5 and CEACAM6 in different lung tissues with respect to COPD, smoking status and granulocyte infiltration, immunohistochemically stained paraffin sections of 19 donors were studied. To address short-term effects of cigarette smoke and acute inflammation, transcriptional regulation of CEACAM5, CEACAM6 and different CEACAM1 isoforms by cigarette smoke extract, interferons, Toll-like receptor agonists, and bacteria was tested in normal human bronchial epithelial (NHBE) cells by quantitative PCR. Corresponding CEACAM protein levels were determined by flow cytometry.

**Results:**

Immunohistochemical analysis of lung sections showed the most frequent and intense staining for CEACAM1, CEACAM5 and CEACAM6 in bronchial and alveolar epithelium, but revealed no significant differences in connection with COPD, smoking status and granulocyte infiltration. In NHBE cells, mRNA expression of CEACAM1 isoforms CEACAM1-4L, CEACAM1-4S, CEACAM1-3L and CEACAM1-3S were up-regulated by interferons alpha, beta and gamma, as well as the TLR3 agonist polyinosinic:polycytidylic acid (poly I:C). Interferon-gamma also increased CEACAM5 expression. These results were confirmed on protein level by FACS analysis. Importantly, also NTHI and *M. catarrhalis* increased CEACAM1 mRNA levels. This effect was independent of the ability to bind to CEACAM1. The expression of CEACAM6 was not affected by any treatment or bacterial infection.

**Conclusions:**

While we did not find a direct correlation between CEACAM1 expression and COPD, the COPD-associated bacteria NTHi and *M. catarrhalis* were able to increase the expression of their own receptor on host cells. Further, the data suggest a role for CEACAM1 and CEACAM5 in the phenomenon of increased host susceptibility to bacterial infection upon viral challenge in the human respiratory tract.

## Background

CEACAMs are members of the immunoglobulin superfamily and are involved in the regulation of various cellular functions such as differentiation, proliferation, apoptosis and many more [1]. All CEA family members are highly glycosylated and mediate homophilic and/or heterophilic binding. CEACAM1 is found on epithelial cells, endothelial cells and on leukocytes, including granulocytes, T cells, B cells, and NK cells [2-4]. CEACAM5 is expressed on epithelial cells and CEACAM6 on granulocytes and epithelial cells [1]. Many CEACAMs can be recruited by different pathogens as receptors and, in addition to effectuate adhesion, mediate bacterial internalization and even evasion of immune responses [3]. Pathogens interacting with members of the CEA family include gram-negative bacteria colonizing human respiratory mucosa, like *Neisseria meningitidis*, typable and non-typable *Haemophilus influenzae* and *Moraxella catarrhalis*[5-11]. The ability of pathogens to target CEACAMs possibly allows them to colonize mucosal surfaces by mediating adhesion and by exploiting immunosuppressive functions of CEACAM receptors. Pathogen binding to CEACAM1 is of especial importance since it leads to the suppression of several receptors that activate important cellular pathways of the innate or adaptive immune response, such as Toll-like receptor (TLR) 2, TLR4, T-cell receptor and B-cell receptor [12-18]. The interferences with TLR signaling as well as the interference with T-cell activation by CEACAM1 depend on the phosphorylation of the immuno-receptor tyrosine-based inhibitory motif (ITIM) contained within the cytoplasmic domain of the long CEACAM1 isoforms [15,16,19].

In human lung epithelia, three members of the CEA protein family known to interact with pathogens are found: CEACAM1, CEACAM5 and CEACAM6 [20-24]. CEACAM5 and CEACAM6 are glycosylphosphatidylinositol (GPI)-anchored to the cell membrane and possess seven and three extracellular Ig-like domains, respectively [24]. The four most commonly co-expressed CEACAM1 isoforms, CEACAM1-4L, CEACAM1-3L, CEACAM1-4S and CEACAM1-3S, possess three or four extracellular Ig-like domains, a trans-membrane domain and a long or a short intracellular domain [24]. While CEACAM1-L is able to transmit signals via its ITIM, CEACAM1-S is still able to mediate homophilic and heterophilic interactions and can trigger actin cytoskeleton re-organization [25,26]. CEACAM1-S can thus not only mediate adhesion as well as internalization of pathogens [27], but can also control the signaling activities of the long CEACAM1 isoforms [28].

Chronic obstructive pulmonary disease (COPD) is a major cause of morbidity and mortality and is expected to be the third leading cause of death, and the fifth leading cause of disability by 2020 [29]. The Global Initiative for Chronic Obstructive Lung Disease defines COPD as characterized by persistent airflow limitation that is usually progressive and associated with an enhanced chronic inflammatory response in the airways and the lung to noxious particles or gases. Exacerbations are associated with increased airway and systemic inflammation, and evidence suggests that 70% may be caused by microorganisms [30]. Two pathogens that are associated with acute exacerbations and the progression of COPD are the CEACAM-interacting pathogens *M. catarrhalis* and *H. influenzae*, which often colonize the mucosa of the lower human respiratory tract in patients with COPD [31-33]. *M. catarrhalis* and *H. influenzae* express structurally unrelated outer membrane proteins, ubiquitous surface protein A1 (UspA1), and P5-homologous adhesin (P5), respectively, that share the ability to bind to the extracellular immunoglobulin V (IgV)-like domain of human CEACAM1 [5,8]. The interaction of CEACAM1 with *M. catarrhalis* results in reduced TLR2-initiated inflammatory responses of primary pulmonary epithelial cells [16]. CEACAM5 and CEACAM6 can mediate bacterial adhesion as well [5,7,8,34]. All three CEACAMs in human airway epithelia can therefore be of importance for the colonization of the lower airways and have a role in acute exacerbations. Since the lower respiratory airways are normally sterile and protected by mucociliary clearance, CEACAMs expressed here are most likely to encounter bacteria in medical conditions leading to dysfunction of the mucociliary clearance, such as COPD [35]. To date, a comprehensive analysis of (co-) expression patterns of CEACAM1 isoforms, CEACAM5 and CEACAM6 in the different lung tissues is lacking.

In the present study, we found CEACAM1, CEACAM5, and CEACAM6 expression on all pulmonary epithelia of the majority of the tested 19 individuals. Expression patterns were not dependent on COPD, smoking status and granulocyte infiltration. In NHBE cells, CEACAM1 expression was enhanced upon exposure to interferons, the TLR3 agonist polyinosinic:polycytidylic acid (poly I:C), *M. catarrhalis*, and NTHi. However, there were no differences in the induction of the different CEACAM1 isoforms tested (CEACAM1-4L, CEACAM1-3L, CEACAM1-4S and CEACAM1-3S). CEACAM5 expression was increased by interferon-gamma and CEACAM6 was unaffected by all treatments tested.

## Methods

### Materials and antibodies

All materials used were from Sigma or Merck unless stated otherwise. Mouse monoclonal antibodies: B3-17 and C5-1X (both mono-specific anti-human CEACAM1, B. B. Singer/Reliatech), 5C8C4 and 2H8-5 (both mono-specific anti-human CEACAM5, B. B. Singer), 13H10 and 1H7-4B (both mono-specific anti-human CEACAM6, Genovac and B.B. Singer), 6/40c (mono-specific anti-human CEACAM8, B.B. Singer), and IgG1 isotype control antibody (Antibodies-Online). Polyclonal antibodies: HRP-coupled rabbit anti-mouse IgG (Dako), HRP-coupled goat anti mouse IgG (Dianova), rabbit polyclonal anti-CEA IgG (Dako), HRP-coupled goat anti-mouse IgG (Dianova), Alexa Fluor 488-conjugated goat anti-mouse IgG (Life Technologies), PE-conjugated goat anti-mouse IgG (H+L, Antibodies-Online).

### Acquisition and processing of human lung specimens

Tissue was obtained from surgical specimens, patients underwent surgery for lung resection to treat lung cancer. A positive vote of the ethics committee of the University of Heidelberg and informed consents were obtained. The resected tissue was fixed in formalin and embedded in paraffin using standard procedures [36].

### Immunohistochemical analysis

Immunohistochemical staining for CEACAM1, CEACAM5, CEACAM6 and CEACAM8 using 20 μg/ml of the monoclonal antibodies B3-17, 5C8C4, 1H7-4B and 6/40c, respectively, was performed on paraffin wax sections obtained from 19 different human lung sections. Sections were deparaffinized (Histoclear 3 × 5 min, ethanol 100% 5 min, ethanol 96% 5 min, ethanol 80% 5 min, ethanol 70% 5 min) and rehydrated (H_2_O 2 × 5 min). Rabbit serum-blocking (2% in PBS) was performed before the sections were incubated with primary antibody over night at 4°C, and with HRP-coupled secondary rabbit antibody for 1 h at RT. Then the nickel-glucose oxidase development technique was performed to enhance the DAB chromogen of the peroxidase immunohistochemistry. The specimens were counterstained with Calcium Red for 1–2 minutes to visualize the tissue structure. The result was a distinct purple/black staining product. As negative control served an IgG1 istotype matched control antibody. Non-cancer tissues from the sections were used for analysis.

### Acquisition of bronchoalveolar lavage fluid (BALF)

BALF samples were obtained from 181 individuals in whom a bronchoscopy was performed for different diagnostic or therapeutic purposes. All individuals underwent bronchoscopy following standard diagnostic procedures at the department of infectious diseases and pulmonary medicine at the Charité -Universitätsmedizin Berlin with informed consents from the patients. The study was approved by the ethical committee of the Charité and all samples were available as residual material without any personal information or clinical data. BALF was centrifuged at 1,200 rpm and supernatants were then collected and stored in aliquots at −80°C until processing for ELISA.

### CEACAM1-, CEACAM5-, and CEACAM6-specific Sandwich-ELISA

Frozen BALF samples were thawed and centrifuged at 2000 × g and 4°C for 10 min. Then CEACAM1, CEACAM5, and CEACAM6 were assessed in the supernatants. 96 well micro titer plates (MaxiSorb TM plates, Nunc) were coated for 2 h at room temperature with 3 μg/ml rabbit anti-CEA-antibody (Dako) diluted in PBS. After washing the plate twice with 0.05% Tween (Carl Roth GmbH) in PBS all unbound sites were blocked for 2 h at room temperature with PBS containing 3% bovine serum albumin (Carl Roth GmbH). To quantify the different CEACAMs in the samples appropriate standard curves were prepared by making serial dilutions of recombinant human CEACAM1-Fc, recombinant human CEACAM6-Fc and purified CEA (from 0.25 ng/ml to 100 ng/ml). The standard and the undiluted samples were incubated over night at 4°C and washed three times. Then 20 μg/ml C5-1X (anti-CEACAM1), 5C8C4 (CEACAM5), or 1H7-4B (CEACAM6) were added. Plates were washed three times with PBS and supplemented with HRP-coupled goat anti mouse antibody (Dianova) for 2 h followed by three washing steps. Then 100 μl TMB-X-tra substrate (Biotrend Chemikalien GmbH) were added and incubated for up to 30 min. The reaction was stopped by 20 μl of 2 N H_2_SO_4_ (Carl Roth) and optical densities were read at 450 nm in a microplate reader (Tecan). All antibodies, samples and standard curves were diluted in PBS containing 1.5 % BSA. The linear ranges for CEACAMs 1, 5, and 6 were 0.25 ng/ml - 100 ng/ml.

### Preparation of aqueous phase cigarette smoke extract (CSE)

CSE was prepared as described [37]. Briefly, smoke from one cigarette (10 mg Tar, 0.8 mg nicotine; Camel Filters, Japan Tobaco International) with the mouthpiece filter removed was bubbled through 10 ml complete medium using a peristaltic pump (P-1, GE Healthcare) and filtered through a 0.22 μm filter. Suction was regulated, so that sidestream smoke developed during the entire combustion, lasting 5 min for each cigarette. The filtered CSE was regarded as 100%. CSE was used within 30 min of preparation at a final concentration of 4%. Lau et al. showed that NHBE cells were induced to secrete IL-8 and remained viable after tratment with 4% CSE for 24 h [37].

### Cell culture, treatments and infections

Primary normal human bronchial epithelial (NHBE) cells (Lonza) were propagated as suggested by the supplier in collagen I-coated flasks or plates (BD Biosciences) in Bronchial Epithelial Cell Basal Medium (BEBM, Lonza) supplemented with bovine pitituary extract (BPE), Hydrocortisone, human Epidermal Growth Factor (hEGF) Epinephrine, Transferrin, Insulin, Retinoic acid and Triiodothyronine (from the BEGM bullet Kit, Lonza). Prior to stimulation, cells in passage 4–6 were grown to confluence. NHBE cells were treated for the indicated durations with 100 ng/ml IFNα (interferon alpha 1a, ImmunoTools), 100 ng/ml IFNβ (interferon beta 1a, ImmunoTools), 100 ng/ml IFNγ (recombinant human interferon gamma, Promokine), 100 ng/ml TNFα (recombinant human tumor necrosis factor alpha, R&D Systems), 100 ng/ml MALP-2 (Enzo Life Sciences GmbH), 100 ng/ml poly I:C (high molecular weight, InvivoGene), 100 ng/ml flagellin (Invivogene Biotech), or 4% CSE (see above). The concentrations chosen for the treatments described above are standard concentrations used for lung epithelial cells [37-43]. Infections werde done using the following strains: *M. catarrhalis*: 25238 (wild type, American Type Culture Collection); BBH18 (wild type) and BBH18.1 (adhesin UspA1 deletion mutant unable to bind CEACAM1), both kindly provided by Kristian Riesbeck, Malmö Lund University, Skåne University Hospital, Malmö, Sweden; non-typable Haemophilus influenzae (NTHi): 2019 (wild type), kindly provided by Edward Swords, University of Iowa, USA; 1128 (wild type) and 1128f- (adhesin P5 deletion mutant unable to bind to CEACAM1), both kindly provided by Lauren O. Bakaletz, The Research Institute at Nationwide Children’s Hospital and The Ohio State University College of Medicine, USA. For infection, bacteria were freshly grown over night at 37°C, 5% CO_2_ on Columbia Agar or Chocolate Agar, respectively (both BD Biosciences). Bacteria were incubated to exponential growth (mid-log phase) in liquid Brain Heart Infusion (BHI) broth at 210 rpm, 37°C (BHI, BD Biosciences; for NTHi supplemented with 10 μg/ml hemin, Sigma Aldrich, and 10 μg/ml NAD, MP Biomedicals). Bacteria were harvested by centrifugation and re-suspended in PBS. Colony forming units (cfu) were determined by measurement of optical densities (M. catarrhalis: OD_405 nm_ = 0.3 correlates with 5×10^7^ cfu/ml; NTHi: OD_600__nm_ = 0.1 correlates with 10^8^ cfu/ml). Cells were infected for 24 h at a multiplicity of infection (MOI) of 5 (*Moraxella catarrhalis*) or 100 (NTHi). Optimal MOIs were determined in pilot experiments by analysis of maximal induction of interleukin 8 secretion in the absence of cytotoxic effects (data not shown).

### Immunoprecipitations from cell culture supernatants

NHBE cell culture supernatants from confluent cells were harvested after 48 h. CEACAM1, CEACAM5 and CEACAM6 were precipitated from 10 ml supernatant using 20 μl protein G-Sepharose 4 Fast Flow (GE Healthcare) and 3 μg of monoclonal antibodies B3-17, 2H8-5, and 13H10, respectively. An IgG1 isotype control was included. Immunoprecipitations were analyzed by Western blot using anti CEA polyclonal antibody that cross-reacts with all three CEACAMs and HRP-coupled goat anti-rabbit IgG. Analysis was done using the Fusion FX System (Peqlab) and SuperSignal West Pico Chemiluminescent Substrate (Thermo Scientific).

### RT-PCR and qPCR analysis

To analyze the gene expression of CEACAM1, CEACAM5, and CEACAM6, total RNA was extracted from 2×10^6^ NHBE cells using the Qiagen RNeasy mini kit. Residual genomic DNA was removed by on-column incubation with DNaseI (Qiagen). A NanoDrop D-1000 Spectrophotometer (Thermo-Fisher Scientific) was then used to assess the amount and quality of the isolated RNA samples. First-strand complementary DNA (cDNA) was synthesized from 2 μg of RNA using the High Capacity cDNA Reverse Transcription Kit (Applied Biosystems). To detect the expression of the CEACAM genes by PCR, specific primers for each target were designed using the on-line primer-BLAST tool of the National Center for Biotechnology Information (NCBI, http://www.ncbi.nlm.nih.gov/tools/primer-blast/). Possible secondary structures at the primer binding sites were taken into account by characterizing the nucleotide sequence of the regions of interest using the Mfold algorithm [44]. Primer design across different exon boundaries allowed for specific primer pairs targeting 7 isoforms of CEACAM1. Primers were also designed for CEACAM5, CEACAM6, two housekeeping genes, and IFNβ-1a.

PCRs of the cDNAs were carried out on a S1000TM Thermal Cycler (BioRad) in a 25 μl reaction volume containing 0.2 μM primers, 1 U Taq DNA polymerase (5-Prime) and 200 μM dNTPs. Thermal conditions included an initial 95°C denaturation step for 3 min, and then 35 cycles of 10 s at 94°C, 30 s at 60°C and 30 s at 72°C. The resulting PCR products were separated on an ethidium bromide containing agarose gel and visualized under a UV-transiluminator to confirm the expected amplicon size. To verify the identity of amplified PCR fragments of the CEACAM genes and the different splicing variants of CEACAM1, PCR-products were purified and sequenced (Eurofins MWG Operon, Ebersberg, Germany). Sequences were aligned with the corresponding NCBI-reference transcripts using ClustalX [45].

To quantify the relative expression of each gene, we used a CAS-1200 pipetting robot (Qiagen) to set up the qPCR-reactions and a Corbett Rotor-Gene 6000 (Qiagen) as Real-Time qPCR apparatus. Each sample was analyzed in duplicate in a total reaction volume of 20 μl containing 10 μl of 2× SensiMix SYBR Master Mix (Bioline) and 0.2 μM of each primer. The cycling conditions included an initial step of 95°C for 10 min followed by 40 cycles of 95°C for 15 s, 60°C for 20 s and 72°C for 20 s. For each experiment, an RT-negative sample was included as a control. Melting curve analysis and size verification by electrophoresis was used to confirm the specificity of the qPCR reactions.

The relative expression of the target genes was analyzed using the Pfaffl method [46]. The expression levels were normalized to the geometric mean of 2 housekeeping genes: hypoxanthine phosphoribosyltransferase1 (HPRT1) and peptidylpropyl isomerase B (PPIB). The stability of the housekeeping genes was assessed using the BestKeeper algorithm [47]. Relative differences in mRNA expression between different experimental conditions were analyzed by pair-wise fixed randomization tests using the REST 2009 software [48].

### Confocal microscopy

NHBE cells were grown on cover slips, fixed in PBS/4% paraformaldehyde and blocked with PBS/10% bovine serum albumin. Confocal images of indirect immunofluorescence of CEACAM1, CEACAM5 and CEACAM6 (B3-17, 2H8-5, and 16H10, respectively, and Alexa Fluor 488-conjugated goat anti-mouse IgG), actin (TRIC-conjugated phalloidin) and nuclei (Hoechst33342) were taken using a Zeiss 710 confocal microscope equipped with the Plan-APO 40X/1.4 Oil objective. Pictures were processed using the Zen Software (Zeiss) and Adobe Photoshop.

### FACS analysis

NHBE cells were cultured and treated as described above, detached using trypsin/EDTA (Invitrogen/Life Science) and kept in PBS/2% FBS on ice. Cells were stained using antibodies B3-17 (CEACAM1) and 2H8-5 (CEACAM5) at 10 μg/ml, and 1:200 PE-conjugated goat anti-mouse IgG. Analysis was done using the FACSAria II (BD Biosciences) and FlowJo (Tree Star Inc.) Software.

## Results

### CEACAM expression in human lung

**Figure 1:**
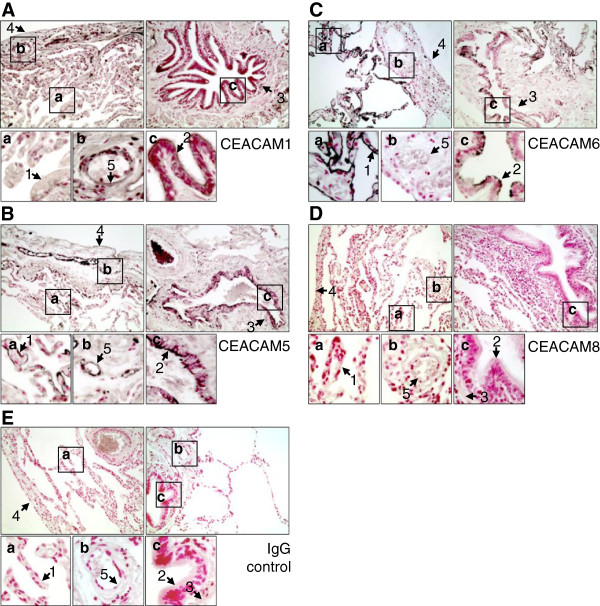
**Expression of CEA family members in human lung.** Paraffin sections of human lung tissue from surgical specimens of lung cancer patients were stained for **(A)** CEACAM1, **(B)** CEACAM5, **(C)** CEACAM6, **(D)** CEACAM8 and **(E)** IgG control antibody using indirect immune-peroxidase technique. Note that blood cells give a positive signal due to intrinsic peroxidase activity. Non-cancer tissues were used for analysis. 1, alveolar epithelium; 2, bronchial epithelium; 3, adventitia submucosa; 4, pleura; 5, pulmonary blood vessel endothelium. Original magnification 200X.

Expression and co-expression patterns of CEACAM1, CEACAM5 and CEACAM6 in the human lung were analyzed. Paraffin sections of lung tissues from 19 donors who underwent surgery for lung resection to treat lung cancer (Table 1) were stained with an IgG control antibody and antibodies specific for CEACAM1, 5 and 6, as well as with an antibody specific for CEACAM8, which is solely expressed in human granulocytes. Non-cancer tissues from these sections were used for analysis. Figure 1 shows representative CEACAM stainings of lung sections for the respective antibodies. Table 2 gives an overview of the number of positive tissues in the specimens. CEACAM1 was expressed in 12 out of 19 lungs, CEACAM5 in 18 out of 19 and CEACAM6 in all 19 lungs examined (Figure 1 and Table 2). As expected, granulocyte-specific CEACAM8 (Figure 1D, Table 2) showed strong staining if infiltrated granulocytes appeared (data not shown) and did not give any staining above IgG background in granulocyte-free lung tissues (Table 2).

**Table 1:** Patient characteristics

Male	9
Female	10
Age	34-80 years
Smoker	6
Chronic obstructive pulmonary disease (COPD)	8
Hypertension	10
Granulocyte infiltration	9

**Table 2:** Analysis of CEACAM expression in human lung sections

	Positive specimen *	**Alveolar epithelium**	**Bronchial epithelium**	**Adventitia submucosa**	**Pleura**	**Blood vessel endothelium**
Control IgG	3^§^/19	1/19	1/15	1/15	1/18	0/18
aCEACAM1	12/19	10/19	10/17	5/17	10/17	11/19
aCEACAM5	18/19	16/19	17/17	11/17	12/17	15/19
aCEACAM6	19/19	19/19	15/18	0/18	6/18	0/19
aCEACAM8	1^§^/19	1/19	0/16	0/16	1/18	0/19

Paraffin sections of human lung tissue from surgical specimens of lung cancer patients (N=19) were stained for CEACAM1, CEACAM5, CEACAM6, CEACAM8 and an unspecific control IgG.

Figure 1 shows representative pictures of non-cancer tissues for these stainings. CEACAM expression in non-cancer tissues was analyzed for alveolar epithelium, bronchial epithelium, adventitia submucosa, pleura and blood vessel endothelium. Since not all tissue types were found for all specimens, the number of positive specimen and the number of total specimen including the respective tissue are given. *Assessment of total staining includes alveolar epithelium, bronchial epithelium, adventitia submucosa, pleura and blood vessel endothelium and excludes present blood cells due to intrinsic peroxidase activity. ^§^Four lung sections showed enhanced background staining for the control IgG or the anti-CEACAM8 antibody.

CEACAM1 expression was most intense in bronchial epithelium, where it was found in 10 specimens out of 19 (Figure 1A, Table 2). Blood vessel endothelium also showed distinct CEACAM1 staining (11/19). A weaker expression was found in alveolar epithelium and pleura (10/19 and 10/17 respectively). In some cases the adventitia submucosa was CEACAM1 positive (5/17).

CEACAM5 displayed frequent and intense staining in bronchial and alveolar epithelium (Figure 1B, Table 2). The former tissue was always positive when found in the respective specimen (17/17), the latter in 16 out of 19 samples. Also, pleura and blood vessel endothelium were often CEACAM5 positive (12/17 and 15/19, respectively). A weaker and less frequent CEACAM5 expression was found in the adventitia submucosa (11/17).

CEACAM6 showed an abundant and strong staining in the alveolar epithelium of all lungs examined (Figure 1C, Table 2). The bronchial epithelium of most specimens also displayed a strong CEACAM6 expression (15/18). In some cases, the pleura exhibited a weak CEACAM6 staining (6/18). Adventitia submucosa and blood vessel endothelium did not show any CEACAM6 expression.

### CEACAM co-expression patterns in human lung tissues

The co-expression patterns of CEACAM1, CEACAM5 and CEACAM6 in the different lung tissues of the 19 specimens were also analyzed (Table 3). In alveolar epithelium, CEACAM6 was present in all specimens; therefore, only CEACAM1 and CEACAM5 expressions varied. CEACAM1 was mostly co-expressed with both, CEACAM5 and CEACAM6. In bronchial epithelium, CEACAM5 was expressed in all specimens; only CEACAM1 and CEACAM6 expression varied. Both proteins were co-expressed with CEACAM5 alone and with each other. The adventitia submucosa was always negative for CEACAM6. CEACAM1 and CEACAM5 were expressed alone or co-expressed. When the pleura was positive for CEACAM6, at least one of the two other CEACAMs were co-expressed. Most of the CEACAM6-negative specimens showed pleura staining for CEACAM5, and some for CEACAM5 and CEACAM1. Blood vessel endothelium was negative for CEACAM6 and showed staining for CEACAM1 and CEACAM5 either alone or together (Table 3). Co-expression patterns showed no correlation to COPD, smoking status and granulocyte infiltration in total staining analysis or in the individual lung tissues (alveolar epithelium, bronchial epithelium, adventitia submucosa, pleura and blood vessel endothelium; data not shown).

**Table 3:** Analysis of CEACAM co-expression patterns in human lung sections

	Positive specimen* (total staining)	**Alveolar epithelium**	**Bronchial epithelium**	**Adventitia submucosa**	**Pleura**	**Blood vessel endothelium**
CC1+/CC5+/CC6+	12	8	7	-	3	-
CC1+/CC5+/CC6-	-	-	3	4	4	8
CC1+/CC5-/CC6-	-	-	-	1	-	3
CC1-/CC5+/CC6+	6	8	7	-	1	-
CC1-/CC5+/CC6-	-	-	-	7	4	7
CC1-/CC5-/CC6-	-	-	-	4	2	1
CC1-/CC5-/CC6+	-	1	-	-	-	-
CC1+/CC5-/CC6+	1	2	-	-	1	-
No evaluation possible^§^	-	-	2	2	4	-

Paraffin sections of human lung tissue from surgical specimens of lung cancer patients (N=19) were stained for CEACAM1, CEACAM5, and CEACAM6. Figure 1 shows representative pictures for these stainings. Non-cancer tissues were analyzed for the co-expression of CEACAM1, CEACAM5 and CEACAM6. Numbers of specimens with the respective pattern denominated in column 1 are given. *Assessment of total staining includes alveolar epithelium, bronchial epithelium, adventitia submucosa, pleura and blood vessel endothelium, and excludes present blood cells due to intrinsic peroxidase activity. §One or more stainings for CEACAM1, CEACAM5 or CEACAM6 are missing for this tissue in a specimen preventing an evaluation.

### CEACAM expression in human lung does not correlate with COPD, smoking status or granulocyte infiltration

It was then examined whether the presence or expression level of CEACAM1, CEACAM5 and CEACAM6 were linked to COPD or smoking status. Table 4 shows the relative staining intensity of the three CEACAMs. Total staining of the paraffin sections, including alveolar epithelium, bronchial epithelium, adventitia submucosa, pleura and blood vessel endothelium, was assessed in the range of 0 (no staining) to 3 (strong staining). However, no correlation was found for either the presence (data not shown) or the expression levels (Table 4) of the tested CEACAMs according to two-tailed Student’s t-test. Next, the interdependency of CEACAM1, CEACAM5 and CEACAM6 expression with granulocyte infiltration was analyzed, but again no correlation was detected (Table 4). Also, the correlation of CEACAM expression with COPD, smoking status and granulocyte infiltration in the individual lung tissues (alveolar epithelium, bronchial epithelium, adventitia submucosa, pleura and blood vessel endothelium) was examined, but no significant differences were found (data not shown).

**Table 4:** No association of CEACAM expression with pathologic conditions in human lung

	**Leukocyte infiltration**		**COPD**		**Smokers**		**Hypertension**	
	**+**	**-**	**+**	**-**	**+**	**-**	**+**	**-**
CEACAM1	0.67 (0.71)	1.00 (1.00)	1,13 (0.67)	0.47 (0.81)	1.00 (0.63)	0.72 (0.83)	0.70 (0.67)	1.00 (0.87)
CEACAM5	1.22 (0.67)	1.90 (1.00)	1.25 (0.92)	1.94 (0.60)	1.50 (0.84)	1.62 (0.77)	1.80 (0.63)	1.33 (0.87)
CEACAM6	2.44 (0.53)	2.70 (1.00)	2.50 (0.53)	2.82 (0.50)	2.50 (0.55)	2.59 (0.51)	2.60 (0.52)	2.56 (0.53)
CEACAM8	0.11 (0.33)	0.10 (0.77)	0.13 (0.32)	0	0	0.07 (0.28)	0	0.11 (0.33)
IgG control	0.11 (0.33)	0.20 (1.14)	0.25 (0.42)	0.06 (0.30)	0.17 (0.41)	0.10 (0.38)	0.20 (0.42)	0.11 (0.33)

Paraffin sections of human lung tissue from surgical specimens of lung cancer patients (N=19) were stained for CEACAM1, CEACAM5, CEACAM6, CEACAM8 and an unspecific control IgG. Figure 1 shows representative pictures for these stainings. Relative total stainings (including alveolar epithelium, bronchial epithelium, adventitia submucosa, pleura and blood vessel endothelium) were assessed in the range of 0 (no staining) to 3 (strong staining). Numbers given are mean intensity and standart deviation. No significant differences were found in comparison to the respective control groups according to two-tailed Student’s t-test.

### Soluble CEACAMs are present in human airways

**Figure 2:**
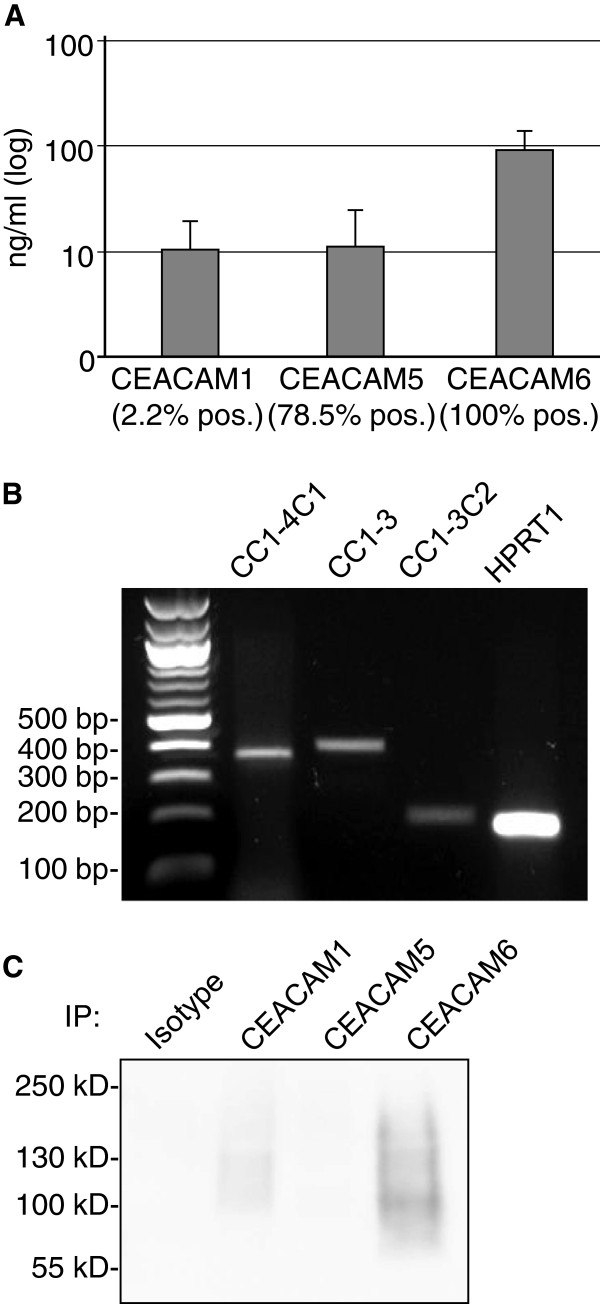
**Soluble CEACAMs in bronchoalveolar lavage fluid and NHBE cells. (A)** Bronchoalveolar lavage fluid from 181 donors was tested for presence of soluble CEACAM1, CEACAM5 and CEACAM6 by colorimetric sandwich ELISA using specific antibodies. The diagram shows mean CEACAM concentrations (ng/ml) and standard error of positive samples (CEACAM1=4, CEACAM5=142 and CEACAM6=181 positive samples; logarythmic scale; SD in the *Results* text). Percentages below columns give percent of BALF samples positive for the respective CEACAM. **(B)** Confluent NHBE cells were tested for the expression of soluble CEACAM1 isoforms CEACAM1-4C1, CEACAM1-3 and CEACAM1-3C2 by RT-PCR. HPRT1 mRNA expression is shown as control housekeeping gene. **(C)** NHBE cell culture supernatants were tested for the presence of soluble CEACAM1 (approx. 110 kDa), CEACAM6 (approx. 90 kDa) and CEACAM5 (approx. 180 kDa) by immunoprecipitation with specific monoclonal antibodies (B3-17, 2H8-5, and 13H10) or an IgG1 isotype control and subsequent Western blot analysis with polyclonal anti-CEACAM5 antibody that cross-reacts with all three CEACAMs.

Next, the presence of soluble CEACAM1, CEACAM5 and CEACAM6 in bronchoalveolar lavage fluid (BALF) was evaluated. Therefore BALF samples were examined by ELISA (Figure 2A). CEACAM1 was found with an average concentration of 10 ng/ml (± 9 ng/ml SD) in 4 out of 181 BALF samples (2.2%). CEACAM5 was present in 78.5% of BALF samples with an average concentration of 11 ng/ml (± 13.6 ng/ml SD) and CEACAM6 in all BALF samples with at an average concentration of 93 ng/ml (± 48 ng/ml SD).

In order to approach soluble CEACAM1 expression in human lung with an alternative method, normal human bronchial epithelial (NHBE) cells from healthy donors were used for transcription analysis. RT-PCR and subsequent sequencing of the obtained PCR-products confirmed the expression of three soluble CEACAM1 isoforms, CEACAM1-4C1, CEACAM1-3 and CEACAM1-3C2 (Figure 2B). However, mRNAs of all three isoforms were not expressed at sufficient levels to allow for reliable quantification analysis by qPCR. To test for expressed and shedded soluble CEACAMs in NHBE cells, CEACAM1-, 5-, and 6- immunoprecipitations from cell culture supernatants were analyzed. Western blot analysis revealed soluble CEACAM6 and low amounts of soluble CEACAM1, but no soluble CEACAM5 (Figure 2C) in NHBE cell culture supernatants.

### CEACAM1 isoforms are up-regulated by interferons and TLR3 agonist poly I:C

In order to investigate the regulation of CEACAMs by cigarette smoke or in the presence of acute inflammation in human lung, NHBE cells were stimulated as described below and the expression of CEACAMs was analyzed using RT-PCR and qPCR. Untreated confluent NHBE cells expressed CEACAM5 and CEACAM6, as well as the CEACAM1 isoforms CEACAM1-4L, CEACAM1-4S, CEACAM1-3L and CEACAM1-3S. This was shown through RT-PCR analysis with pairs of primers specific for each CEACAM and isoforms (Table 5). Identity of the obtained PCR-products for each CEACAM and isoforms was confirmed by sequencing. The intensity of the resulting bands after electrophoretic separation suggests that both CEACAM1 isoforms bearing the long cytoplasmic domain were expressed to a much lesser extent than the two short CEACAM1 isoforms. To address short-term effects of smoking on epithelial CEACAM expression, NHBE cells were exposed to CSE. To assess the tie of CEACAM expression in human lung cells to innate immune responses, NHBE cells were treated with IFNα, IFNβ, IFNγ, TNFα, and the TLR agonists MALP-2 (TLR2/6), poly I:C (TLR3) and flagellin (TLR5). Then, RNA from these cells was isolated and qPCR analysis was used to check for differences in CEACAM1-4L, CEACAM1-3L, CEACAM1-4S, CEACAM1-3S, CEACAM5 and CEACAM6 mRNA levels. To ensure an accurate normalization of their expression levels, we tested two candidate housekeeping genes for their stability across the unstimulated and stimulated samples. PPIB (SD(±Ct)=0.24; CV(%Ct)=1.24) as well as HPRT1 (SD(±Ct)=0.34; CV(%Ct)=1.42) showed a highly stable expression, allowing for normalization with the geometric means of both genes.

**Table 5:** Primers (RT-PCR and qPCR)

**Gene/variant Symbol**	**Forward Primer**	**Reverse primer**	**Size (bp)**
CEACAM1-4L	AAGACGATCATAGTCACTGAGCT	GGAGACTGAGGGTTTGTGCT	483
CEACAM1-4S	AAGACGATCATAGTCACTGAGCT	ATTGGAGTGGTCCTGAGCTG	454
CEACAM1-3L	TCATAGTCACTGATAATGCTCTACC	GGAGACTGAGGGTTTGTGCT	188
CEACAM1-3S	TCATAGTCACTGATAATGCTCTACC	ATTGGAGTGGTCCTGAGCTG	159
CEACAM1-4C1	AAGACGATCATAGTCACTGAGCT	TTGCACACCATTGACAGAGT	369
CEACAM1-3	CAGTGACCCAGTCACCTTGA	TGGACTTGTTTGTGCCTGTTG	403
CEACAM1-3C2	CAAGACGATCATAGTCACTGAGTC	AGAGGGACATATAGGAAGGGGT	210
CEACAM5	AGGCCAATAACTCAGCCAGT	GGGTTTGGAGTTGTTGCTGG	104
CEACAM6	TCAGCCACTGGCCTCAATAG	TCTGGTCCAATCTGCCAGTC	177
HPRT1	GACCAGTCAACAGGGGACAT	AACACTTCGTGGGGTCCTTTTC	195
PPIB	ATGTAGGCCGGGTGATCTTT	TGAAGTTCTCATCGGGGAAG	219
IFNβ-1a	TGCTCTCCTGTTGTGCTTCT	CCACAGGAGCTTCTGACACT	103

Structure of forward and reverse primers (5′-3′) of all target genes and isoforms and the expected size of their respective amplified PCR-products.

**Figure 3:**
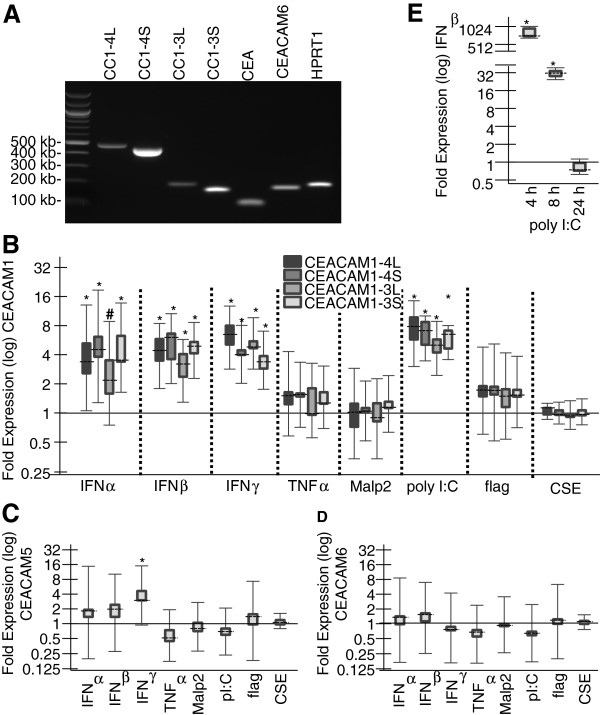
**Regulation of CEACAM expression by CSE, cytokines, and TLR agonists. (A)** Confluent NHBE cells were tested for the expression of CEACAM1-4L, CEACAM1-3L, CEACAM1-4S, CEACAM1-3S, CEACAM5, CEACAM6 and the house-keeping gene HPRT1 by RT-PCR using the primers listed in Table 5. **(B**, **D**, **E)** Confluent NHBE cells were incubated with IFNα, IFNβ, IFNγ, TNFα, MALP-2, poly I:C, flagellin, or CSE, respectively, for 24 h. Real time qPCR was then used to analyze these cells for CEACAM1-4L, CEACAM1-4S, CEACAM1-3L and CEACAM1-3S mRNA expression **(B)**, CEACAM5 mRNA expression **(C)** or CEACAM6 mRNA expression **(D)**, respectively. **(E)** Confluent NHBE cells were incubated with poly I:C for 4, 8 and 24 h and were tested by qPCR for IFNβ mRNA expression. (B-E) The mRNA expression of each transcript was normalized against two housekeeping genes. Expression ratios relative to unstimulated cells were calculated using REST2009 and are represented by box-whisker plots. Dashed lines represent the median expression; boxes and whiskers represent the interquartile range and the maximum and minimum observation, respectively. *P < 0.05, #P = 0.124.

The four transmembrane CEACAM1-isoforms CEACAM1-4L, CEACAM1-3L, CEACAM1-4S and CEACAM1-3S were basically co-regulated by all reagents that elicited a difference in transcription levels (Table 5, Figure 3B). IFNα significantly upregulated the expression of CEACAM1-4L (3.7-fold, p < 0.05), CEACAM1-4S (4.8-fold, p < 0.05) and CEACAM1-3S (4.3-fold, p < 0.001), whereas the 3.5-fold upregulation of CEACAM1-3L failed to reach significance (p = 0.124). IFNβ and IFNγ significantly increased the expression of all four splicing variants of CEACAM1 (p < 0.05 in all cases). In the case of IFNβ we could observe a slightly higher up-regulation of the two short isoforms CEACAM1-4S (5.2-fold) and CEACAM1-3S (4.7-fold) than the long isoforms CEACAM1-4L (4.4-fold) and CEACAM1-3L (3.0-fold). On the other side, IFNγ seemed to favor the two long isoforms CEACAM1-4L (6.3-fold) and CEACAM1-3L (5.2-fold) when compared to the short variants CEACAM1-4S (4.2-fold) and CEACAM1-3S (3.5-fold). However, no significant alteration in the ratios between long and short CEACAM1 isoforms was observed. Poly I:C treatment also up-regulated all four isoforms of CEACAM1 in a significant manner (p < 0.001 in all cases) with expression ratios ranging between 4.9 and 7.0-fold (Figure 3B). CSE, TNFα, MALP-2 and flagellin had no effect on any of the four CEACAM1 transcripts despite of a significant increase of the IL-8 mRNA levels, which served as positive control of their efficacy (data not shown).

In order to examine whether the positive responses of the CEACAM1 isoforms to poly I:C were caused by the up-regulation of interferons, poly I:C treated NHBE cells were analyzed for IFNα, IFNβ and IFNγ mRNA expression. IFNα and IFNγ mRNAs were not expressed at sufficient levels to allow qPCR analysis (data not shown). However, qPCR showed that poly I:C strongly induced an immediate IFNβ expression (780-fold increase after 4 h) that dissolved after 24 h (Figure 3E). qPCR showed that IFNγ treatment also significantly increased CEACAM5 mRNA levels in NHBE cells by 4.1-fold (Figure 3C). No difference in CEACAM5 mRNA levels were induced by CSE, IFNα, IFNβ, TNFα, or any of the TLR agonists. CEACAM6 expression levels in NHBE cells were not altered under any of the tested conditions (Figure 3D).

### Increased CEACAM1 and CEACAM5 mRNA levels lead to increased protein levels on NHBE cell surfaces

**Figure 4:**
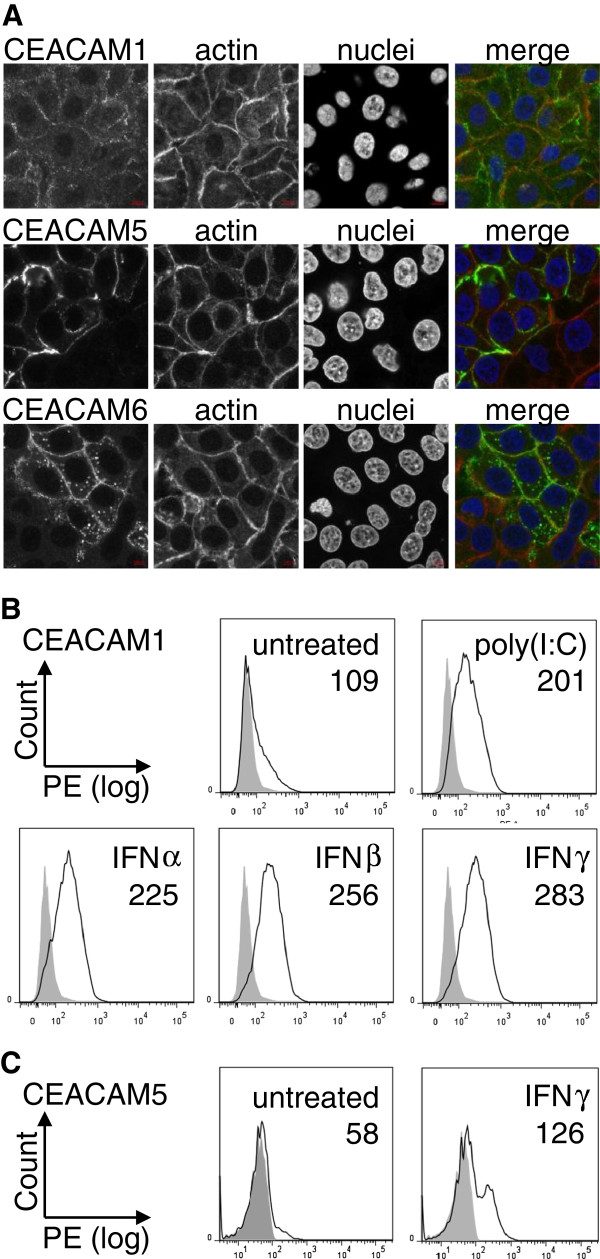
**Cell surface expression of CEACAM1, CEACAM5 and CEACAM6 in NHBE cells. (A)** Confocal images of indirect immunofluorescences of CEACAM1, CEACAM5 and CEACAM6 in non-permeabilized NHBE cells using mono-specific antibodies. Actin was detected using TRIC-conjugated phalloidin and nuclei were stained with Hoechst33342. Merged images display pseudo-colors; green: CEACAMs, red: actin, blue: chromatin. **(B)** FACS analysis of CEACAM1 expression on NHBE cell surfaces. Confluent NHBE cells were left untreated or incubated for 24 h with polyI:C, IFNα, IFNβ or IFNγ, respectively, and subjected to FACS analysis. Data are representative of three experiments with similar results. Filled histograms: IgG control, open histograms: CEACAM1. Numbers give mean intensity (arbitrary units). Mean IgG control = 62. **(C)** FACS analysis of CEACAM5 expression on NHBE cell surfaces. Confluent NHBE cells were left untreated or incubated with IFNγ for 24 h and subjected to FACS analysis. Data are representative of three experiments with similar results. Filled histograms: IgG control, open histograms: CEACAM5. Numbers give mean intensity (arbitrary units). Mean IgG control = 51.

Indirect immunofluorescence showed that CEACAM1, CEACAM5 and CEACAM6 were expressed on the cell surface of untreated confluent NHBE cells (Figure 4A). While CEACAM1 showed a low expression on most cells, CEACAM5 and CEACAM6, respectively, were only expressed by a subpopulation.

It was then tested whether the up-regulation of CEACAM1 transcripts upon treatment with IFNα, IFNβ, IFNγ, and poly I:C were also reflected by higher cell surface protein levels. FACS analysis confirmed an increased expression of CEACAM1 on the cell surface of NHBE cells under all four conditions (Figure 4B). The CEACAM1-specific antibody used for flow cytometry did not allow discrimination between the different isoforms.

Also, an increased CEACAM5 cell surface expression upon IFNγ treatment could be confirmed by FACS analysis (Figure 4C). Flow cytometry also verified that there was only a small subpopulation of NHBE cells that were CEACAM5 positive in untreated control cells. Upon IFNγ treatment, both, the number of CEACAM5-positive cells and the expression level of CEACAM5 on the cell surface, were increased (Figure 4C). However, it remained only a defined portion of NHBE cells that expressed CEACAM5.

### Non-typable *Haemophilus influenzae* (NTHi) and *Moraxella catarrhalis* up-regulate CEACAM1 expression

**Figure 5:**
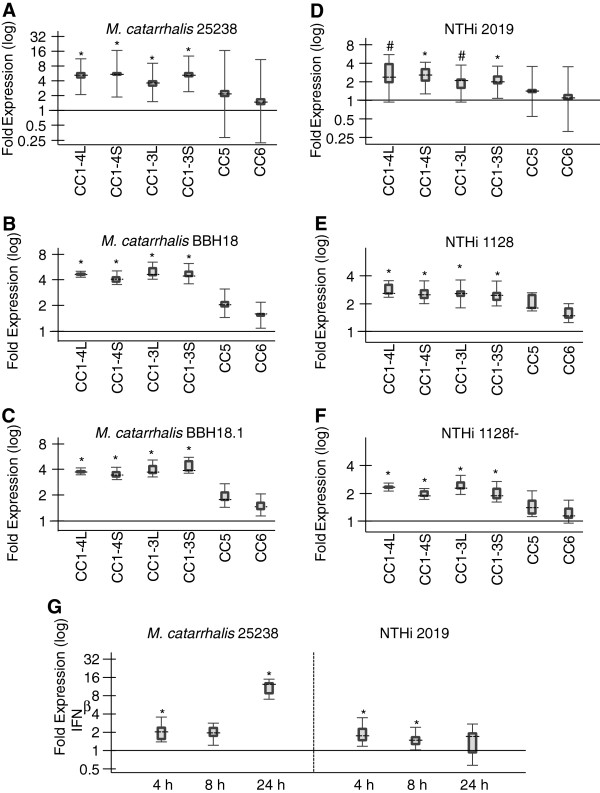
**Regulation of CEACAM expression by M. catarrhalis and non-typable H. influenzae (NTHi).** Confluent NHBE cells were incubated with *M. catarrhalis* strains 25238 (wild type, **A**), BBH18 (wild type, **B**), BBH18.1 (UspA1 deletion mutant unable to bind CEACAM1, **C**), or NTHi strains 2019 (wild type, **D**), 1128 (wild type, **E**), 1128f- (P5 deletion mutant unable to bind to CEACAM1, **F**) for 24 h. Cells were then subjected to qPCR analysis of CEACAM1-4L, CEACAM1-3L, CEACAM1-4S, CEACAM1-3S, CEACAM5, and CEACAM6 mRNA expression. **(G)** Confluent NHBE cells were incubated with *M. catarrhalis* strain 25238 or NTHi strain 2019 for 4 h, 8 h or 24 h and subjected to qPCR analysis for IFNβ mRNA expression. **(A**-**G)** Distrubution of the normalized expression ratios are represented by box-whiskers plots. Dashed lines represent the median expression; boxes and whiskers represent the interquartile range and the maximum and minimum observation, respectively. *P < 0.05, # P = 0.088, §P = 0.079.

Next, the effects of acute NTHi and *Moraxella catarrhalis* infection on CEACAM1, CEACAM5 and CEACAM6 mRNA expression levels in NHBE cells were investigated (Figure 5). qPCR analysis revealed no differences in CEACAM5 and CEACAM6 expression upon bacterial infection. The *M. catarrhalis* wild type strains 25238 and BBH18 as well as the NTHi wild type strains 2019 and 1128 enhanced the mRNA expression of all four transmembrane CEACAM1-isoforms to a similar degree in a co-regulatory manner (Figure 5A,B,D,E). The mean induction of CEACAM1 transcription by *M. catarrhalis* strains was twice as high as by NTHi strains (3.5-5.5 fold vs. 1.9-2.8 fold). Since all four pathogens can bind to CEACAM1, we next tested whether this interaction was essential to the up-regulation of CEACAM1. To that end we used the *M. catarrhalis* UspA deletion mutant BBH18.1 and the NTHi P5 deletion mutant 1128f-, which both lack the respective CEACAM1-binding adhesin (Figure 5C,F). Again, the infection with these strains induced an elevated CEACAM1 expression (4.0-4.9 fold and 1.9-2.4 fold, respectively) comparable to their parental strains, indicating a CEACAM1-independent, more general mechanism for this effect. We then tested whether the CEACAM1 up-regulation might be due to an increase in interferons. Both *M. catarrhalis* 25238 and NTHi 2019 induced only a very small increase in IFNβ mRNA levels in NHBE cells that in part were not significant (Figure 5C). IFNβ mRNA levels were elevated two-fold or less after 4 and 8 h by both pathogens (compared to the 780-fold increase by poly I:C). However, *M. catarrhalis* induced as a secondary effect a significant 10.9-fold increase in IFNβ mRNA levels after 24 h.

## Discussion

Here we present the first comprehensive study based on immunohistochemistry demonstrating that CEACAM1, CEACAM5, and CEACAM6 are frequently co-expressed in several tissues of the human lung, including epithelia of the airways and alveoli. CEACAM expression was not connected to COPD, smoking status and granulocyte infiltration (Figure 1, Tables 3 and 4). Despite the analysis of non-cancer tissues from the specimen, the fact that the lung sections used for immunohistochemical analysis were from patients that underwent lung resection to treat lung cancer might conceal a regulatory effect of COPD or smoking status on CEACAM expression, since CEACAM1, CEACAM5, and CEACAM6 have all been shown to be up-regulated in lung cancer [49-53]. Also, the inflammatory processes associated with cancers of the lung might have had an effect on the expression levels of the CEACAMs. For example, as discussed below, IFNγ can up-regulate CEACAMs 1, 5, and 6. Importantly, we show that the COPD-associated pathogens *M. catarrhalis* and NTHi can also upregulate CEACAM1 expression independent of their ability to bind to CEACAM1.

The up-regulation by *M. catarrhalis* might be at least in part due to the induction of IFNβ production (Figure 5G) and is in accordance with the observation of CEACAM1 up-regulation by pathogenic *Neisseria* in endothelial and epithelial cells [54,55]. CEACAM5 and CEACAM6 expression levels were not affected by *M. catarrhalis* and NTHi. However, both receptors were already expressed at high levels and in all specimens (Figure 1, Tables 2 and 3). Regarding colonization, the increase in CEACAM1 on epithelial cells upon bacterial challenge is likely to increase bacterial adherence and infection. This interaction would be facilitated by an impaired mucocilliary clearance, which is associated with later phases of COPD. Thus, CEACAM receptors might be at least in part responsible for the colonization of the lower airways by *M. catarrhalis* and NTHi in COPD patients. This setting would also explain the association of bacterial colonization with progressive/advanced disease despite the fact that we did not find a regulation of CEACAM expression by the chronic pathologic conditions COPD, smoking status and granulocyte infiltration (Figure 1, Tables 3 and 4). Further studies including identification of pathogens in the lower airways will be necessary to shed light on this aspect of CEACAM-pathogen interactions. It will be also important to take into account that an up-regulation of CEACAMs might be transient either due to temporary qualities of chronic/persistent disease or due to pathogen characteristics at distinct time points during the infection process. It will also be interesting to test human lung tissues for the presence of the other CEACAMs expressed on epithelial cells (CEACAM7, 18, 20 and 21 [56,57]) and to analyze their ability to interact with pathogens.

The up-regulation of membrane-bound CEACAM receptors might be necessary to counteract the presence of soluble receptor forms that might act as decoys to prevent bacterial/viral infections or immune evasion. Even though bacteria possess redundant targeting mechanisms, and bacterial adhesins often work in a sequential manner, Hill et al. showed that perturbing CEACAM1-, CEACAM5- and CEACAM6-based adhesion by using a CEACAM-binding peptide prevents host cell binding by *M. catarrhalis*, *H. influenzae*, *N. meningitidis* and *N. gonorrhoeae* in a cell culture model [7]. The amounts of soluble CEACAMs, with high concentrations of soluble CEACAM6 in all and lower concentrations of soluble CEACAM5 in 78.5% of the bronchoalveolar lavage fluid samples is mirrored in the expression levels of their membrane-bound counterparts in lung tissues (Figures 1, 2, 3, 4 and [58-60]). The fact that soluble CEACAM1 is all but absent in BALF (98% negative BALF samples) increases the importance of this receptor for colonization by pathogens despite its comparatively low expression levels in human lung.

In NHBE cells, CEACAM1 mRNA and protein levels were also increased by IFNα, IFNβ, IFNγ and the TLR3 agonist poly I:C (Figure 3B). All membrane-bound CEACAM1 isoforms examined here were basically co-regulated under all conditions that affected their expression level (Figures 3 and 5). However, some differences were found for the differential up-regulation of the CEACAM1 isoforms. Type I interferons (IFNα and IFNβ) favored the two short isoforms while the type II interferon IFNγ favored the long isoforms, and poly I:C favored the long and the short isoforms bearing 4 extracellular domains. Especially the former differences, even though not significant, are interesting with regard to the fact that in order to interfere with TLR signaling or with T-cell activation, binding of the Shp-1 phosphatase to the immunoreceptor tyrosine-based inhibitory motif (ITIM) within the cytoplasmic domain of the long CEACAM1 isoform was necessary [15,16,19]. The two tyrosine residues that are part of the ITIM can bind to protein tyrosine kinases (e.g. c-Src) as well as protein tyrosine phosphatases (e.g. Shp-2) - leading to the stimulation or the inhibition of cell-signaling pathways, respectively. Long and short CEACAM1 isoforms can appear as monomers, dimers and oligomeric microclusters in the membrane [26,28]. Trans-homophilic binding between different CEACAM1 molecules increases cis-dimerization in the plane of the membrane via an allostery-based mechanism. Binding of CEACAM1-L to Shp-1 or c-Src is dependent on the balance between the oligomeric states as well as the ratio of long and short isoforms [26,28]. Interestingly, no cytoplasmic domain is necessary for the CEACAM1-mediated internalization of *H. influenzae*, *M.catarrhalis* and *N. gonorrhea*[27]. But bacterial engagement of CEACAM1 might, in addition to procuring adherence, also influence the cis-dimerization and subsequent inhibitory or activatory signaling of CEACAM1, either supporting pathogen colonization or host response. For example, the interaction of CEACAM1 with *M. catarrhalis* and *N. meningitidis* proteins resulted in reduced Toll-like receptor (TLR) 2-initiated inflammatory responses (the major mediator of *M. catarrhalis*-induced immune responses) of NHBE cells [16,61,62].

Part of the host response to microbial infection is the production of cytokines [63]. Type I interferons IFNα and IFNβ can be produced by most cell types and are the major effector cytokines of the host immune response against viral infections. However, the production of type I interferons is also induced in response to bacterial infections [64]. The type II interferon IFNγ plays an important role during the immune response to bacterial pathogens, but is also induced upon infection with viruses. It is produced predominantly by natural killer (NK) cells and natural killer T cells as part of the innate immune response and by Th1 CD4+ and CD8+ cytotoxic T lymphocyte effector T cells once antigen-specific immunity develops [65]. In the present study CEACAM5 is up-regulated in NHBE cells by IFNγ, but not by type I interferons, and CEACAM1 is increased by type I and type II interferons (IFNβ and IFNγ, Figure 3B and D). While for CEACAM1, CEACAM5 and CEACAM6 an up-regulation by IFNγ and viral infections has been described in epithelial cells [55,66-70], to our knowledge this is the first report that also type I interferons enhance CEACAM1 expression.

A temporal association between bacterial and viral infections is often observed in the human upper respiratory tract [71,72]. Infection by opportunistic colonizers, including *Haemophilus influenzae* and *Moraxella catarrhalis*, increases considerably following influenza and/or respiratory syncytial virus (RSV) infections [71,72]. COPD is often associated with viral infections, mostly by rhinovirus, and it was recently shown that these infections indeed precipitate secondary bacterial infections, particularly in COPD patients [73].

Importantly, the present study shows that in addition to the primarily viral induced type I interferons, TLR3 agonist poly I:C also increased CEACAM1 expression (Figure 3B). TLR3 plays a key role in anti-viral immune responses and recognizes synthetic dsRNA like poly I:C and virus derived dsRNA contained in cells infected by positive-stranded RNA viruses and DNA viruses [74,75]. It was shown recently that poly I:C enhances the susceptibility to secondary pulmonary infections by gram-positive bacteria in a mouse model [76]. The positive-stranded RNA virus rhinovirus enhances CEACAM5 expression in human nasal epithelial cells and two negative-stranded RNA viruses, respiratory syncytial virus (RSV) and human parainfluenza virus 3 (HPIV-3), enhance CEACAM1 expression in A549 and NHBE cells [67,77]. Since the latter viruses have to be recognized via TLR7 or TLR8, this is the first indication that the pathogen receptor CEACAM1 might also be up-regulated by positive-stranded RNA viruses via TLR3.

CEACAM6 expression was not altered by any agent used in this investigation (Figure 3D). This was probably due to its initially high expression level in the NHBE cells. Fahlgren *et al.* showed that the LS174T cell line that expressed high levels of CEACAM5 and CEACAM6 before IFNγ treatment did not show any enhanced expression after IFNγ exposure while IFNγ up-regulated both mRNAs in two cell lines, HT-29 and T84, that initially expressed low levels of CEACAM5 and CEACAM6 [66]. However, the very high and constant CEACAM6 expression in NHBE cells as well as in the lung specimens is in accordance with its proposed role as a surfactant [58,78].

The up-regulation via viruses and the effects of the inflammatory cytokine IFNγ imply a more general role for CEACAM1 and CEACAM5 in the inflammatory response to infection, and also in the spatial and temporal association between bacterial and viral infections. One of the underlying mechanisms for the colonization of COPD patients by *M. catarrhalis* and NTHi may consist of the up-regulation of specific host receptors, i.e. CEACAM1, by viral infections, as described for several CEA family receptors [55,66,68,77]. For pathogenic *Neisseria* it was demonstrated that increased CEACAM expression levels correlated with an increase in bacterial invasion [55,79]. Once established, *M. catarrhalis* and NTHi themselves are also able to increase the expression of their receptor CEACAM1. Whether the starting point is a viral or bacterial pathogen, the result is a continual cycle of infection-induced increase in cytokine levels and, subsequently, receptor expression which then promotes bacterial invasion. Since viruses also recruit CEACAMs, elevated CEACAM expression might aid viral infection as well [80,81].

## Conclusions

CEACAM1 is a well-established receptor for bacteria, including the human pathogens *M. catarrhalis* and *H. influenza* that both colonize about one third of all COPD patients and cause acute exacerbations. While we did not find a direct correlation between CEACAM expression and COPD, these COPD-linked bacteria were able to increase the expression of their own receptor on host cells. We also propose a role for the constitutively expressed CEACAM6 as well as CEACAM1 and CEACAM5 in the phenomenon of increased host susceptibility to bacterial infection upon viral challenge in the human respiratory tract, as it occours for example in COPD patients, since either enhanced CEACAM1 expression induced by viruses or the COPD-associated changes in the function of the mucociliary clearance that will allow an easy access to CEACAMs on respiratory epithelia are likely to increase bacterial colonization.

## Abbreviations

CEACAM: Carcinoembryonic antigen-related cell adhesion molecule; COPD: Chronic obstructive pulmonary disease; HPRT1: Hypoxanthine phosphoribosyltransferase1; IFN: Interferon; ITIM: Immunoreceptor tyrosine-based inhibitory motif; M. catarrhalis: *Moraxella catarrhalis*; NHBE: Normal human bronchial epithelial cells; NTHI: Non-typable *Haemophilus Influenzae*; poly I:C: Polyinosinic:polycytidylic acid; PPIB: Peptidylpropyl isomerase B; TLR: Toll-like receptor.

## Competing interests

The authors declare that they have no competing interests.

## Authors’ contributions

HS conceived, designed and coordinated the study. EK analyzed immunohistochemical pictures and performed statistical analysis. TEK, EK and IS designed, performed and analyzed qPCR experiments. MMM and EK performed and analyzed immunoprecipitations, Western blots, and confocal microscopic studies. BBS did immunohistochemical experiments and performed and analyzed ELISAs. AH performed and analyzed FACS experiments. EK, TEK and KAH accomplished NHBE stimulations. HD, CG and RB collected lung specimen and performed paraffin embedding and sectioning. EK and HS drafted the manuscript. All co-authors read and approved the final manuscript.
